# Редкие формы наследственных новообразований эндокринной системы: аденома гипофиза в сочетании с феохромоцитомой и/или параганглиомой

**DOI:** 10.14341/probl13196

**Published:** 2023-05-11

**Authors:** Е. О. Мамедова, Д. В. Лисина, Ж. Е. Белая

**Affiliations:** Национальный медицинский исследовательский центр эндокринологии; Национальный медицинский исследовательский центр эндокринологии; Национальный медицинский исследовательский центр эндокринологии

**Keywords:** аденома гипофиза, феохромоцитома, параганглиома, синдромы множественных эндокринных неоплазий, сукцинатдегидрогеназа, MAX

## Abstract

Гормонально-активные аденомы гипофиза, так же как и феохромоцитомы/параганглиомы, встречаются в общей популяции достаточно редко. При этом аденомы гипофиза в рамках наследственных синдромов составляют до 5% всех случаев аденом гипофиза, тогда как феохромоцитомы/параганглиомы могут быть наследственными в 30–40% случаев. К наследственным синдромам, ассоциированным с аденомами гипофиза, относят синдромы множественных эндокринных неоплазий 1 и 4 типов, семейные изолированные аденомы гипофиза, Карни комплекс. Наследственных синдромов, ассоциированных с феохромоцитомами/параганглиомами, и генов, мутации в которых приводят к их развитию, значительно больше. Клинические описания сочетания у одного пациента аденомы гипофиза и феохромоцитом/параганглиом начали появляться с середины ХХ в., однако выделить такое сочетание в отдельный синдром (синдром «3PAs» (pituitary adenoma, pheochromocytoma, paraganglioma)) было предложено лишь в 2015 г. К настоящему времени в литературе описано немногим более 100 случаев такого сочетания, при этом чаще всего в качестве генетической причины синдрома «3PАs» выявляются мутации в генах, кодирующих субъединицы сукцинатдегидрогеназного комплекса II (SDHx), реже выявляются мутации в генах MAX, MEN1 и ряде других. В настоящем обзоре литературы приводятся современные данные о синдроме сочетания аденом гипофиза и феохромоцитом/параганглиом.

## Abstract (en)

Functioning pituitary adenomas and pheochromocytomas/paragangliomas are rare in the general population. Pituitary adenomas occur in the familial setting in approximately 5% of cases, whereas pheochromocytomas/paragangliomas can be hereditary in 30–40% of cases. Hereditary syndromes associated with pituitary adenomas include multiple endocrine neoplasia types 1 and 4, familial isolated pituitary adenomas, and Carney complex. Hereditary syndromes associated with pheochromocytomas/paragangliomas and genes, mutations in which predispose to their development, are more numerous. The first clinical descriptions of the co-occurrence of pituitary adenoma and pheochromocytoma/paraganglioma in one patient date back to the mid 20th century, however delineating such a co-occurrence into a particular syndrome («3PAs» (pituitary adenoma, pheochromocytoma, paraganglioma)) was suggested only in 2015. To date, approximately 100 cases of such a co-occurrence have been described in the literature. Mutations in genes encoding subunits of succinate dehydrogenase complex II (SDHx) are revealed in the majority of cases, much less common are mutations in MAX, MEN1 and some other genes. This review summarizes the current information on the «3PAs» syndrome.

## ВВЕДЕНИЕ

Гормонально-активные аденомы гипофиза (АГ) встречаются в общей популяции достаточно редко, при этом среди спорадических гормонально-активных АГ преобладают пролактиномы (распространенность 50:100 000) [1], далее следуют соматотропиномы (6:100 000) [2] и кортикотропиномы (4:100 000) [3]; другие виды гормонально-активных АГ встречаются реже. Распространенность феохромоцитом (ФХЦ) и параганглиом (ПГ), по данным одного из европейских исследований, составляет 6,4:100 000 [4].

АГ в большинстве случаев являются спорадическими. Наследственные синдромы, ассоциированные с развитием АГ, встречаются в ~5% случаев [5]. К последним относятся: синдром множественных эндокринных неоплазий (МЭН) 1 типа (вследствие мутаций в гене MEN1), синдром МЭН 4 типа (CDKN1B), семейные изолированные АГ (AIP в некоторых случаях, в большинстве случаев мутации неизвестны), Карни комплекс (PRKAR1A), а также редкие случаи бластом гипофиза при мутациях в гене DICER1 [5][6]. В отличие от АГ, ФХЦ/ПГ являются наследственными в 30–40% случаев [7]. Наследственные синдромы, ассоциированные с ФХЦ/ПГ, подразделяют на 1 типа (SDHD), 2 типа (SDHAF2), 3 типа (SDHC), 4 типа (SDHB), 5 типа (SDHA) (далее в тексте статьи эти гены могут быть обобщенно обозначены как SDHx), а также вследствие мутаций в генах TMEM127, MAX, FH, EGLN2 (PHD1), MDH2 и некоторых других [8–10]. Наследственные ФХЦ/ПГ возникают также в рамках других синдромов: МЭН 2 типа (RET), фон Гиппеля–Линдау (VHL), при нейрофиброматозе 1 типа (NF1), редко в рамках синдрома МЭН 1 типа (МЭН-1) [9, 10].

В целом, с учетом небольшой распространенности в популяции обеих патологий, наследственное сочетание гормонально-активных АГ и ФХЦ/ПГ представляется крайне редким.

Синдром «3РАs» — сочетание аденом гипофиза и феохромоцитом/параганглиом (pituitary adenoma, pheochromocytoma/paraganglioma).

Впервые клиническое сочетание АГ (соматотропиномы) и ФХЦ было описано в 1952 г. [11]. Первый случай с доказанной генетической причиной такого сочетания (мутация в гене SDHC у пациента с макропролактиномой и ПГ шеи) был описан в 2008 г. [12]. Далее в 2012 г. была описана мутация в гене SDHD у пациента с агрессивной соматотропиномой, двусторонними ФХЦ и множественными ПГ [13]. В целом сочетание АГ и ФХЦ/ПГ, по мнению зарубежных авторов, теоретически может объясняться следующими причинами: 1) мутация в гене, предрасполагающем к развитию ФХЦ/ПГ, которая приводит также к образованию АГ; 2) мутация в гене, предрасполагающем к развитию наследственных АГ, которая также приводит к образованию ФХЦ/ПГ; 3) дигенное заболевание, т.е. наличие мутаций в двух генах у одного пациента или в одной семье, приводящих к развитию обоих заболеваний; 4) мутация в одном, возможно, новом гене, приводящая к развитию обоих заболеваний; 5) эктопическая продукция гормонов гипоталамуса ФХЦ/ПГ, приводящая к увеличению гипофиза и имитирующая АГ; 6) развитие АГ и ФХЦ/ПГ у одного пациента или в одной семье вследствие случайного спорадического сочетания [14]. В 2015 г. J. Dénes и соавт. описали когорту из 39 пациентов с сочетанием АГ и ФХЦ/ПГ (как спорадические (19 человек), так и семейные случаи (20 человек из 8 семей)). В некоторых семейных случаях АГ и ФХЦ/ПГ были у разных членов семьи. При проведении секвенирования генов, ответственных за развитие ФХЦ/ПГ, известных на тот момент (SDHA, SDHB, SDHC, SDHD, SDHAF2, RET, VHL, TMEM127, MAX, FH), и генов, ответственных за развитие семейных аденом гипофиза (MEN1, AIP, CDKN1B), было выявлено 11 герминальных мутаций (5 SDHB, 1 SDHC, 1 SDHD, 2 VHL и 2 MEN1) и 4 варианта с неопределенной клинической значимостью (2 SDHA, 1 SDHB и 1 SDHAF2) [14]. Авторы обратили внимание, что характерной особенностью АГ у пациентов с мутациями в генах SDHx являлось наличие интрацитоплазматических вакуолей. Авторы обобщили полученные ими данные с данными, опубликованными на тот момент в литературе (70 случаев с 1952 г., суммарно 109 случаев), и выявили, что в 20 случаях были выявлены мутации в генах SDHx (2 SDHA, 8 SDHB, 2 SDHC, 8 SDHD), при этом АГ были представлены пролактиномами, соматотропиномами и гормонально-неактивными АГ. Также среди 109 пациентов с сочетанием АГ и ФХЦ/ПГ были описаны мутации в гене RET (5 пациентов (2 соматотропиномы, 2 пролактиномы, 1 гормонально-неактивная АГ)), в гене VHL (2 пациента (одна соматотропинома и одна АГ смешанной секреции (пролактин и гормон роста)), в гене MEN1 (6 пациентов (5 ФХЦ, 1 ПГ головы и шеи)). На основании полученных данных авторы пришли к выводу, что мутации в генах SDHx могут приводить к образованию АГ, а мутации в гене MEN1 могут приводить к образованию ФХЦ/ПГ [14].

В 2015 г. Р. Xekouki и соавт. предложили термин «синдром 3PAs» (pituitary adenoma, pheochromocytoma, paraganglioma) для обозначения сочетания АГ и ФХЦ/ПГ у пациентов с мутациями в генах SDHx [15]. В этом исследовании среди 22 пациентов с семейными АГ у 4 также имелись ФХЦ и/или ПГ, при этом у 3 из 4 (75%) были выявлены мутации в генах SDHx (1 SDHD и 2 SDHB) [15]. Во всех случаях спорадического сочетания АГ и ФХЦ/ПГ мутации в генах SDHx выявлены не были. Обобщая полученные данные с опубликованными ранее, авторы подтвердили, что АГ чаще представлены соматотропиномами, пролактиномами или гормонально-неактивными АГ, чаще макроаденомами [15]. Также в своей работе авторы провели исследование гипофизов у мышей Sdhb+/- и обнаружили, что гипофизы у таких мышей гиперплазированы, в них повышено количество лактотрофов и соматотрофов. При электронной микроскопии были выявлены морфологические нарушения митохондрий, а также была выявлена повышенная экспрессия фактора, индуцируемого гипоксией 1-альфа (HIF-1a). Таким образом, авторы предположили, что состояние псведогипоксии, возникающее при мутациях в генах SDHx, может запускать каскад реакций, приводящих к опухолевой трансформации клеток аденогипофиза [15]. Также в 2015 г. была опубликована обзорная статья S. O’Toole и соавт., суммировавшая данные о 72 опубликованных случаях сочетания АГ и ФХЦ/ПГ [8]. При этом у 29% из них были найдены мутации в генах, предрасполагающих к развитию семейных ФХЦ/ПГ или АГ (MEN1, RET, SDHB, SDHC, SDHD, SDHAF2, SDHA+VHL), у 32% отмечен отягощенный семейный анамнез и 39% были единичными случаями без подтвержденной мутации [8]. В 2018 г. была опубликована обзорная статья Р. Xekouki и соавт., в которой были суммированы данные о 82 опубликованных случаях сочетания «3PАs» [10]. У 31 пациента (37,8%) из этой когорты имелись мутации в генах, предрасполагающих к ФХЦ/ПГ или АГ. У 22 пациентов (26,8%) имелись данные о положительном семейном анамнезе, что было подозрительно на наличие наследственного эндокринного синдрома, тогда как 37 случаев (45,1%) были единичными случаями в семье; для оставшихся 28% информация о семейном анамнезе отсутствовала. Среди 82 описанных в литературе случаев в 17% имелись и выявленная мутация, и положительный семейный анамнез. Среди пациентов с выявленным генетическим дефектом очевидно преобладали мутации в генах SDHx (19 из 31, 61,3%), мутации в генах MEN1 и MAX были второй и третьей самой частой причиной соответственно [10].

Далее мы рассмотрим отличительные особенности сочетания АГ и ФХЦ/ПГ в зависимости от выявляемых мутаций.

## ГЕНЫ СУКЦИНАТДЕГИДРОГЕНАЗНОГО КОМПЛЕКСА (SDHx)

Сукцинатдегидрогеназа (SDH) — это белок с двойной функцией: он играет роль в цикле Кребса (или трикарбоновых кислот), окисляя сукцинат до фумарата, а также образует комплекс II в митохондриальной респираторной цепи. Комплекс SDH состоит из 4 субъединиц (SDHA, SDHB, SDHC и SDHD) и четырех факторов сборки (SDHAF1, SDHAF2, SDHAF3, SDHAF4) [16]. Гидрофильные белковые субъединицы A и B образуют каталитическое ядро фермента и содержат сайт связывания субстрата для сукцината, в то время как гидрофобные субъединицы C и D закрепляют комплекс на внутренней митохондриальной мембране как митохондриальный комплекс II [16]. Нарушение функции сукцинатдегидрогеназного комплекса приводит к накоплению сукцината, который ингибирует пролилгидроксилазы, которые не могут гидроксилировать фактор транскрипции HIF-1a, что приводит к транскрипции HIF-чувствительных генов и к состоянию тканевой псевдогипоксии, что, в свою очередь, ведет к опухолеобразованию за счет активации ангиогенеза, метаболизма глюкозы и выживания клеток [16]. Мутации в генах SDHx встречаются в около половине случаев наследственных ФХЦ/ПГ [9].

SDHA

Ген SDHA расположен на хромосоме 5p15.33 и кодирует субъединицу А сукцинатдегидрогеназного комплекса (по структуре является флавопротеином) [17]. В 2010 г. была описана герминальная гетерозиготная мутация в гене SDHA у пациентки с ПГ и было доказано, что SDHA, так же как и другие SDHx гены, является геном-супрессором опухолевого роста [18]. Мутации в гене SDHA (так же, как и в генах SDHC и SDHAF2) встречаются реже, чем мутации в генах SDHB и SDHD у пациентов с ФХЦ/ПГ. У пациентов с мутациями в гене SDHA чаще встречаются ПГ, чем ФХЦ, часто отсутствует семейный анамнез заболевания; характерна низкая пенетрантность [19].

В литературе описана одна семья с сочетанием АГ и ФХЦ/ПГ с мутацией в гене SDHA: ПГ у матери и гормонально-неактивная макроаденома гипофиза у сына, с потерей иммуногистохимического окрашивания на SDHA в обеих опухолях [20].

SDHB

Ген SDHB расположен на хромосоме 1p36.13 и кодирует субъединицу В сукцинатдегидрогеназного комплекса (по структуре является железо-серным белком) [21]. В 2001 г. было доказано, что герминальные гетерозиготные мутации в гене SDHB являются причиной наследственных ФХЦ и ПГ [22], при этом чаще всего ПГ множественные и склонны к малигнизации [23]. В литературе описано 12 пациентов с сочетанием «3РАs» и мутацией в гене SDHB [14][15][24–27]. Среди АГ преобладали пролактиномы (как макро-, так и микроаденомы), также встречались соматотропиномы и гормонально-неактивные аденомы. В большинстве случаев встречались ПГ головы и шеи (в том числе злокачественные), в одном случае была выявлена ФХЦ. В ряде случаев описаны пациенты с изолированными АГ и мутациями в гене SDHB, при этом у родственников первой линии родства также имелись ПГ [14][28–30]. Также описан один случай карциномы гипофиза у пациентки с мутацией в гене SDHB и ПГ в анамнезе [31].

SDHC

Ген SDHC расположен на хромосоме 1q21 и кодирует субъединицу С сукцинатдегидрогеназного комплекса [32]. В 2000 г. было доказано, что мутации в гене SDHC являются причиной наследственных ПГ [33]. В литературе описано два случая пациентов с синдромом «3РАs» и мутациями в гене SDHC [12, 14]. В обоих случаях имелись макропролактиномы и ПГ головы и шеи.

SDHD

Ген SDHD расположен на хромосоме 11q23 и кодирует субъединицу D сукцинатдегидрогеназного комплекса [34]. В 2000 г. было доказано, что мутации в гене SDHD являются причиной наследственных ФХЦ и ПГ [34]. SDHD был первым среди SDHx генов, ассоциированным с ФХЦ/ПГ. В литературе описано 6 случаев пациентов с синдромом «3РАs» и мутациями в гене SDHD [13][35–38]. В трех случаях были выявлены макропролактиномы, в двух — макросоматотропиномы, в одном — гормонально-неактивная макроаденома гипофиза. У всех пациентов были выявлены ПГ (преимущественно головы и шеи, но также брюшной полости и таза), в трех случаях они сочетались с ФХЦ [13][35–38].

SDHAF2

Ген SDHAF2 расположен на хромосоме 11q13.1 и кодирует один из факторов сборки сукцинатдегидрогеназного комплекса [39]. В 2009 г. было доказано, что мутации в гене SDHAF2 являются причиной наследственных ПГ [39]. В литературе описан один пациент с макросоматотропиномой и ПГ головы и шеи и вариантом в 5’UTR регионе гена SDHAF2 [14].

В целом следует отметить, что среди носителей мутаций в генах SDHx у пациентов с мутациями в гене SDHB самый высокий риск развития злокачественных ФХЦ/ПГ, у пациентов с мутациями в гене SDHD самая высокая пенетрантность [40]. При мутациях в генах SDHx, помимо ФХЦ/ПГ и АГ, могут возникать гастроинтестинальные стромальные опухоли (ГИСТ, gastro-intestinal stromal tumors, GIST) и почечно-клеточный рак. Следует отметить, что при исследовании большой когорты случаев АГ мутации в генах SDHx практически не выявлялись (0,3%) [41].

## МУТАЦИИ В ГЕНЕ MAX. СИНДРОМ МНОЖЕСТВЕННЫХ ЭНДОКРИННЫХ НЕОПЛАЗИЙ 5 ТИПА?

Ген MAX расположен на хромосоме 14q23.3 и кодирует MYC-ассоциированный фактор Х, который является транскрипционным фактором типа лейциновой молнии и членом семейства белков MYC/MAX/MSD, участвующих в клеточной пролиферации, дифференцировке и апоптозе [7]. В 2011 г. было показано, что мутации в гене MAX предрасполагают к развитию наследственных ФХЦ/ПГ, при этом опухоли расположены в брюшной полости/забрюшинном пространстве, преобладает норметанефриновый тип секреции, в 50% случаев опухоли двусторонние (синхронные или асинхронные), в 40% случаев выявляется положительный семейный анамнез [7][9]. В 2012 г. N. Burnichon и соавт. описали когорту пациентов с мутациями в гене MAX и ФХЦ/ПГ, где у одного пациента также была выявлена АГ (без указания подробностей) [42]. Также на этой когорте было показано, что у носителей мутаций могут развиваться другие опухоли (рак молочной железы, онкоцитома почки, рак почки, плоскоклеточный рак языка) [42]. В 2017 г. K. Roszko и соавт. впервые описали клинический случай сочетания пролактиномы и двусторонней ФХЦ [43], а в 2018 г. A. Daly и соавт. представили еще три случая сочетания АГ и ФХЦ вследствие мутаций в гене MAX [44]. В последующем у одного члена этой семьи также была выявлена нейроэндокринная опухоль поджелудочной железы [45]. Пятый случай был описан A. Kobza и соавт. в 2018 г. [46]. В 2020 г. A. Seabrook и соавт. описали две семьи с мутациями в гене MAX, которые были примечательны не только наличием АГ и ФХЦ/ПГ (злокачественных в некоторых случаях), но и наличием множества других опухолей (паравертебральная ганглионеврома, нейробластома, множественные аденомы околощитовидных желез, хондросаркома, мультифокальный рак легких) [47]. Это позволило авторам выдвинуть предположение, что ген MAX может быть новым геном, ответственным за развитие МЭН, и предложили термин синдрома МЭН 5 типа [47]. Нами также был описан случай сочетания АГ (пролакто-соматотропиномы) и двусторонних асинхронных ФХЦ у пациентки с анамнезом семейной акромегалии [48]. Отмечаются некоторые клинические особенности пациентов с синдромом «3РАs» и мутациями в гене MAX. Среди АГ встречаются как макро-, так и микроаденомы, при этом по типу секреции выявлялись только пролактиномы и соматотропиномы. У большинства пациентов выявлялись двусторонние ФХЦ (как синхронные, так и асинхронные, иногда множественные). В работе А. Seabrook и соавт. был описан случай акромегалии неясной этиологии (либо вследствие АГ, либо вследствие ПГ, продуцирующей гормон роста — рилизинг-гормон) [47].

## ДРУГИЕ НАСЛЕДСТВЕННЫЕ СИНДРОМЫ

Синдром МЭН-1 возникает вследствие мутаций в гене MEN1 и характеризуется развитием опухолей околощитовидных желез, поджелудочной железы, АГ, а также опухолей надпочечников [49]. ФХЦ были описаны у пациентов с генетически подтвержденным синдромом МЭН-1 в менее чем 1% случаев [49], при этом в литературе описано лишь 4 пациента с генетически подтвержденным МЭН-1 и сочетанием АГ и ФХЦ/ПГ, помимо других МЭН-1-ассоциированных опухолей [14]. По типу АГ были представлены пролактиномами или соматопролактиномами, в трех случаях имели место ФХЦ и в одном — ПГ в брюшной полости [14]. В одном из этих случаев в ткани ФХЦ были доказаны потеря гетерозиготности в локусе MEN1 и отсутствие иммуногистохимического окрашивания на менин, что позволило сделать вывод о роли менина в развитии ФХЦ [14]. Кроме того, было описано еще несколько пациентов с МЭН-1 и ФХЦ/ПГ, но без АГ [8].

Синдром МЭН 2А и 2В типов (МЭН-2) возникает вследствие мутаций в гене RET и характеризуется развитием медуллярного рака щитовидной железы, ФХЦ, опухолей околощитовидных желез, а также марфаноподобной внешности и ганглионевром слизистых при МЭН-2В [50]. Было описано два случая сочетания АГ и ФХЦ/ПГ при мутации в гене RET, в обоих случаях АГ были гормонально-активными (кортикотропинома и соматотропинома), однако анализ тканей АГ не проводился [51][52]. Еще в одном случае с доказанной мутацией в гене RET имелась АГ (соматотропинома), но без ФХЦ/ПГ, при этом анализ ткани АГ также не проводился [53]. Таким образом, случаи АГ в рамках МЭН-2 были описаны, однако роль RET в развитии АГ до сих пор не доказана.

Синдром фон Гиппеля–Линдау возникает вследствие мутаций в гене VHL и характеризуется развитием гемангиобластом центральной нервной системы, гемангиом сетчатки, кист или рака почек, кист или нейроэндокринных опухолей поджелудочной железы, а также ФХЦ/ПГ [54]. АГ не являются компонентом синдрома, однако в литературе описан случай развития агрессивной смешанной соматопролактиномы у 15-летнего пациента с генетически доказанным синдромом [55]. В ткани опухоли не было выявлено потери гетерозиготности в локусе VHL [14]. С учетом частоты обследования ЦНС у пациентов с синдромом фон Гиппеля–Линдау отсутствие выявления АГ в большинстве случаев свидетельствует, скорее, о возможном случайном сочетании.

В 2020 г. описан клинический случай сочетания акромегалии и ПГ шеи у 61-летней женщины с вариантом неопределенного значения в интроне 2 гена TMEM127 c245-10C>G [56]. Ген TMEM127 расположен на хромосоме 2q11.2, описан в 2005 г., и мутации в нем выявлены при семейных ФХЦ и ПГ [57].

Случаев сочетания АГ и ФХЦ/ПГ у пациентов с мутациями в гене CDKN1B (синдром МЭН 4 типа (МЭН-4)), мутациями в гене AIP (семейные изолированные аденомы гипофиза (FIPA)), у пациентов с нейрофиброматозом 1 типа (мутации в гене NF1) описано не было.

Краткое описание наследственных синдромов, ассоциированных с сочетанием АГ и ФХЦ/ПГ, представлено в таблице 1.

**Table:** Таблица 1. Наследственные синдромы, в рамках которых могут возникать сочетания АГ и ФХЦ/ПГ (адаптировано из 58 и 59) Сокращения: АД — аутосомно-доминантное; АД* — аутосомно-доминантное, отцовское наследование; + — редко; ++ — часто; +++ — очень часто.

Ген	Хромосома	Синдром	Другие клинические проявления	Наследование	Злокачественные ФХЦ/ПГ	Локализация
Односторонняя ФХЦ	Двусторонние ФХЦ	ПГ таза, брюшной полости, грудной клетки	ПГ головы и шеи	Множественные ПГ
SDHD	11q23	Семейные параганглиомы 1 типа	Гастроинтестинальные стромальные опухоли (+), рак почки (+)	АД*	+	+	-	++	+++	+++
SDHAF2	11q13.1	Семейные параганглиомы 2 типа	-	АД*	-	-	-	-	+++	++
SDHC	1q21	Семейные параганглиомы 3 типа	Гастроинтестинальные стромальные опухоли (++), рак почки (+)	АД	+	+	-	+	++	+
SDHB	1p36.1	Семейные параганглиомы 4 типа	Гастроинтестинальные стромальные опухоли (++), рак почки (+++)	АД	+++	++	+	+++	++	++
SDHA	5p15	Семейные параганглиомы 5 типа	Гастроинтестинальные стромальные опухоли (+++), рак почки (+)	АД	-	-	-	+	+	-
TMEM127	2q11	-	Рак почки	АД	-	+++	++	+	+	+
MAX	14q23.3	-	Рак молочной железы, онкоцитома почки, рак почки, плоскоклеточный рак языка, паравертебральная ганглионеврома, нейробластома, множественные аденомы околощитовидных желез, хондросаркома, мультифокальный рак легких	АД*	+	++	++	+	-	-
MEN1		МЭН-1	Опухоли околощитовидных желез, нейроэндокринные опухоли поджелудочной железы, опухоли надпочечников и др.	АД	-	+	-	+	-	-
RET	10q11.2	МЭН-2А МЭН-2В	Медуллярный рак щитовидной железы, опухоли околощитовидных желез. Для МЭН-2А — амилоидоз кожи Для МЭН-2В — невриномы слизистых, марфаноподобная внешность	АД	-	++	++	-	-	-
VHL	3p25-26	Синдром фон Гиппеля–Линдау	Гемангиобластомы центральной нервной системы, гемангиомы сетчатки, светлоклеточный рак почки, кисты и нейроэндокринные опухоли поджелудочной железы	АД	+	++	+++	+	+	+

## ЗАКЛЮЧЕНИЕ

Молекулярные механизмы сочетанного развития эндокринных опухолей в целом до сих пор остаются малоизученными. Синдром сочетания АГ и ФХЦ/ПГ (синдром «3PAs») является относительно недавно описанным состоянием (по сравнению с классическими синдромами МЭН-1 и МЭН-2). Как показал анализ опубликованных в литературе случаев, чаще вклад в сочетанное развитие АГ и ФХЦ/ПГ вносят гены, ответственные за развитие семейных ФХЦ/ПГ (SDHx, MAX, TMEM127), тогда как среди генов, ответственных за развитие семейных АГ, только для MEN1 была доказана его роль в развитии обоих типов опухолей. Каким образом мутации в генах, ассоциированных с развитием ФХЦ/ПГ, могут приводить к развитию АГ, каскад каких реакций запускается при этом и каким образом можно объяснить тканевую специфичность развития этих опухолей, в настоящее время неясно.

Таким образом, при наличии ФХЦ/ПГ, особенно при наличии отягощенного семейного анамнеза, следует обратить внимание на любые симптомы, которые могут указывать на гиперсекрецию гормона роста или пролактина, а также на нарушение зрения, поскольку большинство АГ в контексте синдрома «3PАs» являются макроаденомами, секретирующими пролактин или гормон роста, или гормонально-неактивными аденомами. Некоторые критерии, такие как более молодой возраст начала симптомов, двусторонняя локализация опухолей и положительный семейный анамнез, могут указывать на наличие наследственного синдрома. Генетическая верификация диагноза целесообразна с применением технологий высокопроизводительного секвенирования (секвенирование панелей, содержащих все известные гены, ассоциированные с ФХЦ/ПГ и АГ, либо полноэкзомное секвенирование). Несмотря на выявление ряда генетических причин сочетания «3PAs», причина развития значительного количества случаев остается невыясненной, что позволяет предположить, что новые гены, ассоциированные с синдромом «3PAs», еще предстоит идентифицировать.

## ДОПОЛНИТЕЛЬНАЯ ИНФОРМАЦИЯ

Источники финансирования. Поисково-аналитическая работа и подготовка статьи проведены за счет гранта Российского научного фонда (проект №19-15-00398).

Конфликт интересов. Авторы декларируют отсутствие явных и потенциальных конфликтов интересов, связанных с содержанием настоящей статьи.

Участие авторов. Мамедова Е.О., Лисина Д.В. — поиск источников литературы, написание текста статьи; Белая Ж.Е. — редактирование, одобрение финальной версии рукописи.

Все авторы одобрили финальную версию статьи перед публикацией, выразили согласие нести ответственность за все аспекты работы, подразумевающую надлежащее изучение и решение вопросов, связанных с точностью или добросовестностью любой части работы.
